# Potential Biomarkers to Distinguish Type 1 Myocardial Infarction in Troponin-Elevated Diseases

**DOI:** 10.3390/ijms24098097

**Published:** 2023-04-30

**Authors:** Sohyen Kwon, Sang-Hyun Park, Sora Mun, Jiyeong Lee, Hee-Gyoo Kang

**Affiliations:** 1 Department of Senior Healthcare, Graduate School, Eulji University, Uijeongbu 11759, Republic of Korea; 2 Department of Internal Medicine, School of Medicine, Eulji University, Daejeon 34824, Republic of Korea; 3 Department of Biomedical Laboratory Science, College of Health Sciences, Eulji University, Uijeongbu 11759, Republic of Korea

**Keywords:** type 1 myocardial infarction, elevated cardiac troponin, proteomics, mass spectrometry

## Abstract

Classifying myocardial infarction by subtype is crucial for appropriate patient management. Although troponin is currently the most commonly used biomarker, it is not a specific marker for myocardial infarction and cannot distinguish subtypes. Furthermore, previous studies have confirmed that proteins known as myocardial infarction markers could function to distinguish the type of myocardial infarction. Therefore, we identify a marker that can distinguish type 1 myocardial infarction from other diseases with elevated troponin. We used mass spectrometry to compare type 1 myocardial infarction with other conditions characterized by troponin elevation and identified new candidate markers for disease classification. We then verified these markers, along with those already known to be associated with cardiovascular disease and plaque rupture. We identified *α*-1 acid glycoprotein 2, corticosteroid-binding globulin, and serotransferrin as potential distinguishing markers. The presence of these markers and other parameters, such as chest pain, electrocardiogram, and troponin levels from the complementary diagnostic processes, could provide valuable information to specifically diagnose type 1 myocardial infarction.

## 1. Introduction

Over eight million patients with signs of acute myocardial infarction (MI) visit hospitals annually [1]. Emergency rooms focus a large amount of attention on these patients [2] because early diagnosis and treatment are critical for their prognoses [3]. Troponin is currently the most effective biomarker for diagnosing MI [4]. The troponin test allows patients to be rapidly diagnosed with myocardial damage with high sensitivity and low imprecision [5,6]. With technological advances, high-sensitivity cardiac troponin (hs-cTn) could help to diagnose patients who would remain undiagnosed using the previous method because of its lower detection limits [5,7]. However, as troponin elevation suggests damage to cardiac cells, it has also been detected in several other diseases [8]. Troponin levels vary depending on the disease. Thus, distinguishing between different diagnoses is challenging [6,9,10,11].

Myocardial infarction is universally pathophysiologically classified into types 1 and 2 [12]. Type 1 MI indicates a necrotic state that causes an oxygen supply imbalance in the cardiac muscle owing to atherothrombotic plaque disruption or erosion in the blood vessels. These can narrow the blood vessels or cause complete occlusion. In contrast, type 2 MI is induced by an imbalance in oxygen supply or demand, rather than thrombotic plaque disruption.

As treatment and management strategies differ based on the MI type [12,13], previous studies have attempted to find new distinct markers based on the type of MI (excluding troponin). Johannes et al. [2] used 29 biomarker panels to identify markers that can distinguish type 1 MI, type 2 MI, and myocardial injury. In this study, myocardial injury is defined as an elevated concentration of the cardiac biomarker troponin in an absence of acute myocardial ischemia. Thomas et al. [14] identified markers that can distinguish type 2 MI from MI using hs-cTn T, hs-cTn I, and 17 cardiovascular biomarkers. Yu et al. [15] assessed the diagnostic efficacy of six biomarkers, either alone or in combination, to distinguish between type 1 MI and type 2 MI. Previous studies also attempted the identification of markers for MI subtype classification by comparing type 1 MI with type 2 MI or MI with myocardial injury. However, to distinguish one specific type of MI using biomarkers, we must consider comparisons of one type of MI with other diseases that may be suspected in the diagnostic process [8].

**Figure 1:**
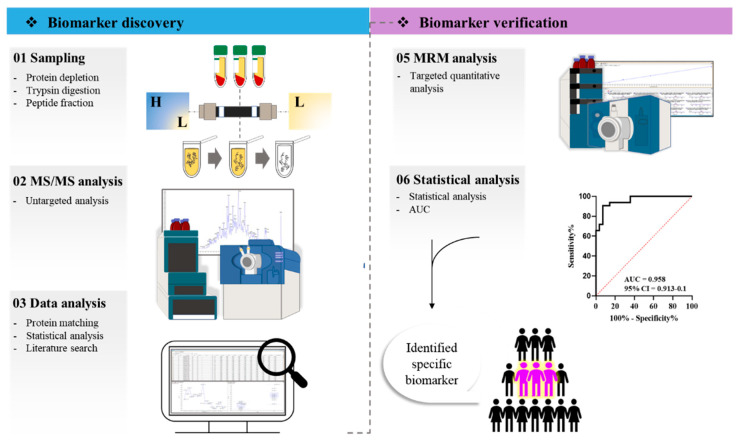
Experimental flow chart. The experiment consisted of biomarker candidate-discovery and verification steps. In the discovery step, serum samples were pretreated, and the entire protein was screened using an untargeted mass spectrometry approach. The final candidates were selected through statistical analysis and a literature search. In the verification step, proteins were selected as the final markers through multiple reaction monitoring (MRM) quantitative analysis and statistically verified.

The aim of this study is to identify markers that can distinguish type 1 MI among various diseases in which troponin is elevated. We identified a new marker using a mass spectrometry (MS)-based approach that analyzed both type 1 MI (the “T1MI” group) and other disorders in which troponin levels are elevated (the “Others” group) (Figure 1). Thrombus and plaque formation are well-established causes of type 1 MI. Thus, in this study, we hypothesized that variables related to the composition or generation of plaque and thrombus could serve as indicators for type 1 MI.

## 2. Results

### 2.1. Patient Characteristics

In this study, parameters from 60 patients were evaluated. Among them, 32 patients were diagnosed with type 1 MI (T1MI), and 28 were classified as Others. For statistical analysis, the discovery set compared 12 patients from the T1MI group against 10 patients from the Others group. The patients were chosen randomly. While all subjects were analyzed for the verification set.

The study subjects had a median age of 73 (interquartile range: 60.5–80.0), with 37 men (61.7%). There were no significant differences in the age or sex distributions between the two analysis sets. In the discovery set, peak troponin (T1MI vs. Others; *p* = 0.014), and creatine kinase MB isoenzyme (CK-MB) (T1MI vs. Others; *p* = 0.009) had greater levels in the T1MI group. Within the verification set, statistically significant differences were observed in the current smoker (T1MI vs. Others; *p* = 0.043), including electrocardiogram (ECG) results for ST-depression (T1MI vs. Others; *p* = 0.02), ST-elevation (T1MI vs. Others; *p* = 0.007), and nonspecific changes (T1MI vs. Others; *p* < 0.0001), as well as in both heart disease biomarkers, troponin (T1MI vs. Others; *p* = 0.002) and CK-MB (T1MI vs. Others; *p* = 0.002). Excluding nonspecific changes in the ECG, higher values were observed in all parameters among patients with type 1 myocardial infarction than among those in the Others group (Table 1). The Others group consisted of patients diagnosed with four types of diseases, and among the causes of type 2 MI, O_2_ imbalance occurred in several preceding diseases (Supplementary Table S1).

### 2.2. Finding Type 1 Myocardial-Infarction-Specific Protein Markers

**Figure 2:**
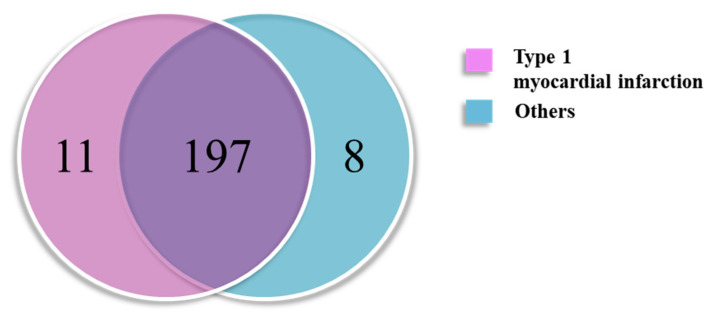
Venn diagram of matched proteins in the type 1 myocardial infarction and Others groups. Others: diseases with elevated cardiac troponin other than type 1 myocardial infarction.

**Figure 3:**
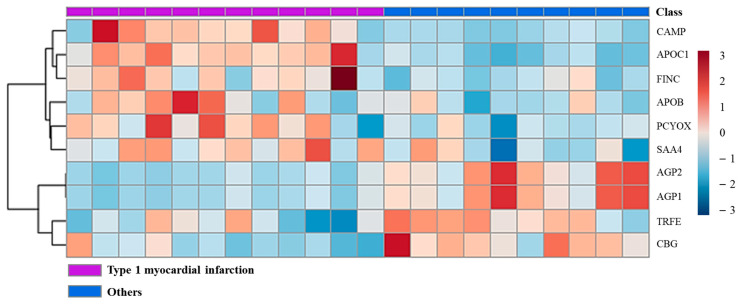
Hierarchical clustering heatmap of candidate proteins. The heatmap depicts correlations according to the expression levels between the group and protein candidates and between the related proteins. Columns represent type 1 myocardial infarction (purple) and Others (blue). Rows represent scaled protein intensity values. Red = high intensity, Blue = low intensity. Others: diseases with elevated cardiac troponin other than type 1 myocardial infarction. Abbreviations: CAMP, cathelicidin antimicrobial peptide; APOC1, apolipoprotein C-I; FINC, fibronectin; APOB, apolipoprotein B-100; PCYOX, prenylcysteine oxidase 1; SAA4, serum amyloid A-4 protein; AGP1, α-1-acid glycoprotein 1; AGP2, α-1-acid glycoprotein 2; TRFE, serotransferrin; CBG, corticosteroid-binding globulin.

The 216 proteins from the data-independent acquisition (DIA) data were matched to the spectral library (Figure 2), including 208 in the T1MI group and 205 in the Others group. Among them, the marker selection process was conducted with 208 proteins that belong to a subset of group A and to the intersection of group B. Moreover, 29 of them met the criteria (*p* < 0.05 and 0.77 ≤ fold change or fold change ≥ 1.3) in the *t*-test results (Supplementary Table S2). Furthermore, the complement group of the general immune system [16] was excluded from the final biomarker candidates. The association between the selected proteins and type 1 MI was confirmed from the literature. Consequently, 10 proteins became candidate markers for verification. A heatmap analysis was performed on the discovered proteins. The increase or decrease in protein expression in each disease group can be visually confirmed (Figure 3).

### 2.3. Quantitative Comparison of Protein Groups Differentiating Type 1 Myocardial Infarction

**Figure 4:**
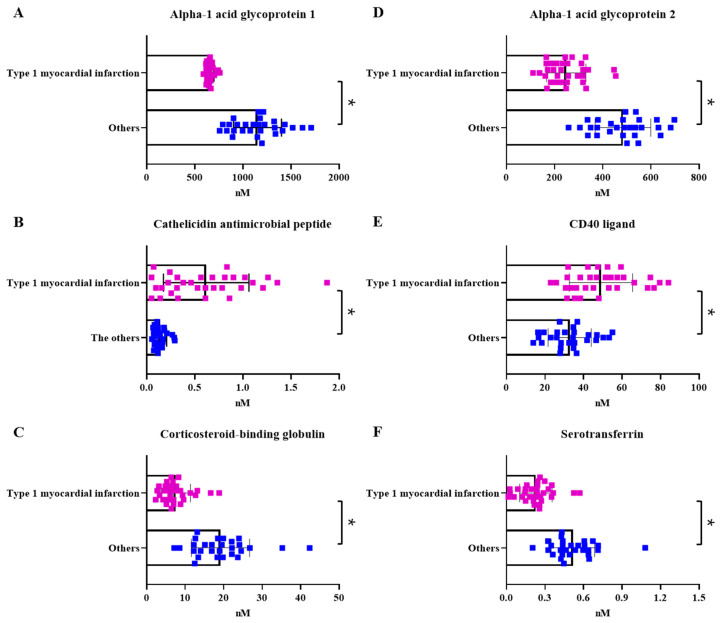
Scatter bar graph. Quantitative protein levels in the comparative groups. (**A**–**F**) Quantitative scatter plot with a bar graph depicting significant comparative group differences. * *p* < 0.0001. Others: diseases with elevated cardiac troponin other than type 1 myocardial infarction.

The peptides were synthesized to a minimum purity of 94%. All calibration curves for each peptide have a coefficient of determination (R^2^) > 0.99 (except for apolipoprotein B-100; R^2^ = 0.95). The quantitative values of thirteen of the fourteen proteins, including the four proteins based on the literature (CD 40 ligand (CD40L), matrix metallopeptidase 9 (MMP9), myeloperoxidase (MPO), and pregnancy-associated plasma protein A (PAP-PA)) that are reportedly related to cardiac artery disease and plaque rupture and are known as markers of MI, were obtained using the MRM final parameter method. The peptide-dependent ion transition and parameters used in the final method were summarized (Table 2). Matrix metallopeptidase 9 was included from the references but was not detected in this study samples. Comparing the quantitative data in the two groups, excluding outliers (ROUT method; Q = 1%), revealed a significant difference in six proteins: α-1 acid glycoprotein 1 (*p* < 0.0001), α-1 acid glycoprotein 2 (*p* < 0.0001), cathelicidin antimicrobial peptide (*p* < 0.0001), CD40 ligand (*p* < 0.0001), corticosteroid-binding globulin (*p* < 0.0001), and serotransferrin (*p* < 0.0001) (Figure 4).

### 2.4. Comparison with the Healthy Control Group and Marker Selection

**Figure 5:**
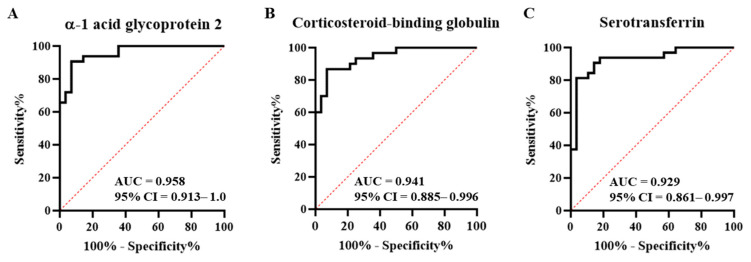
Receiver-operating characteristic (ROC) curve. Confirmation of the marker’s diagnostic capacity. (**A**) The area under the curve (AUC) of α-1 acid glycoprotein 2 is 0.958. (**B**) The AUC of corticosteroid-binding globulin is 0.941. (**C**) The AUC of serotransferrin is 0.927. CI, confidence interval.

In the ROC curve, when evaluating the performance of each biomarker in disease diagnosis, if the area under the curve (AUC) value was >0.9, it showed excellent diagnostic ability [17]. Therefore, three proteins with an AUC value of >0.9 were selected as final markers for diagnosing type 1 MI among elevated troponin diseases (Figure 5). Each marker exhibited high sensitivity and specificity. In AGP2, the sensitivity was 90.63 (75.8–96.8) and the specificity was 89.3 (72.8–96.3). Corticosteroid-binding globulin had a sensitivity of 86.7 (70.3–94.7) and a specificity of 85.7 (68.5–94.3). Serotransferrin had a sensitivity of 84.4 (68.3–93.1) and a specificity of 85.7 (68.5–94.3). In addition, cutoff values of AGP2, CBG, and TREA were <337.3 nM, <12.4 nM, and <0.4 nM, respectively. The cutoff values, sensitivity, and specificity of each marker are listed in Table 3.

Comparison of the healthy control group (a group without cardiovascular disease) with the disease group revealed the altered expression patterns of protein markers (Supplementary Table S3). In most cases, the proteins previously distinguished in each disease exhibited statistically distinct differences between the type 1 MI and healthy groups at the same time (Supplementary Figure S1).

## 3. Discussion

In the present study, we investigated biomarkers, excluding troponin, to distinguish type 1 MI from other diseases with elevated cardiac troponin. The nontargeted approach of mass spectrometry is suitable for finding potential new biomarkers because it can screen the proteins contained in the sample without prior information about them [18,19]. In addition, multiple reaction monitoring can be used to verify quantitative changes in the internal proteins associated with pathophysiology at low cost and without the use of antibodies [20,21]. We discovered novel markers between type 1 MI and other diseases with elevated troponin, including type 2 MI, using mass spectrometry, and then verified their potential as biomarkers. We identified three biomarkers, α-1 acid glycoprotein 2 (AGP), corticosteroid-binding globulin (CBG), and serotransferrin (TRFE), which detected type 1 MI effectively.

Thrombus and plaque provoke type 1 MI. Therefore, in this study, factors involved in the composition or production of plaque and thrombus were hypothesized to work as specific type 1 MI markers. Conditions of inflammation, oxidative stress, and hypoxia lead to plaque disruption, and the plaque and thrombus causal relationship promotes thrombus formation [22,23]. In the thrombus composition, fibrin forms the most abundant part, and platelets, erythrocytes, cholesterol crystals, and leukocytes compose the rest [24].

α-1 acid glycoprotein 2, an acute-phase protein, can be a marker of a clinical condition [25]. AGP mainly synthesizes in the liver [26] but can be detected in the myocardium [27]. This protein has a low concentration in a normal state but is significantly increased in acute-phase conditions caused by inflammatory cytokines, such as glucocorticoids, CCAAT/enhancer binding proteins, interleukin (IL)-1, tumor necrosis factor-α, and IL-6, and counteracts inflammation [28,29]. After MI, the immune system is activated to restore the necrotic lesion, resulting in a severe inflammatory response and high AGP2 levels in the MI [30]. In this study, AGP2 was significantly higher in the Others group. This result may be attributed to various pathophysiological conditions and disorders, but further investigations are warranted for verification [31]. Moreover, the comparison between the disease groups detected differentially expressed protein values. Therefore, AGP2 could be a biomarker to distinguish type 1 MI.

The CBG and TRFE were effective markers that distinguished type 1 MI from other disorders, and both exhibited a distinct decrease compared with their levels in the healthy controls. 

Corticosteroid-binding globulin is primarily synthesized in the liver [32] but can also be found in the heart [33]; however, the tissue origin of CBG is unknown. CBG has a cardinal function that binds and carries glucocorticoids and regulates free hormones [32,34]. Over 90% of cortisol binds to CBG, and the rest binds to albumin or free cortisol [35,36]. CBG has recently attracted attention for its control of free cortisol levels [37]. Glucocorticoids are upregulated to respond to stressors and restore homeostasis, which improves the immune system, secondary metabolism, and cardiovascular function [38]. Notably, only free corticosteroids, which do not bind to CBG, have biological activity under clinical conditions (the free hormone hypothesis) [39]. During inflammation, the cleaved CBG reduces its affinity with cortisol, and the free-hormone emission is increased [37,40]. CBG is an acute negative protein with low expression during inflammation [41], which may be caused by synthesis degradation owing to a reduced affinity with cortisol [42]. Our results align with the previous literature and confirm the increased free cortisol and decreased CBG levels in MI [43]. Here, the distinct decrease in type 1 MI compared with that in the healthy controls could suggest a severe state of type 1 MI [44].

Serotransferrin is a negative acute-phase protein with a decreased tendency in inflammation, tissue necrosis, malignancy, and hepatic dysfunction [45,46]. A commonly recognized transferrin deficiency indicates iron overload and iron deposition can be generated by excessive iron saturation and organization in the transferrin binding site [47]. Ferritin showed a higher level in premature acute MI than in healthy controls, and we can assume the iron level will increase in acute MI [48]. Iron homeostasis plays a vital role in heart function, and iron deficiency and overload have been associated with the causes of heart-related diseases [49,50]. In a previous study, iron overload accelerated reactive oxygen species production, which increased oxidative stress in the blood vessels and consequently accelerated thrombus in rats [51]. In addition, increased oxidative stress with higher serum iron levels can lead to the formation of tighter fibrin networks and induce the formation of atherosclerotic plaques, as well as the reactive and activated state of circulating platelets in MI [52,53,54]. Therefore, the decrease in TRFE indicates an excess of iron and that the induced responses may contribute to thrombogenesis, which affects the generation of type 1 MI.

Previous studies have confirmed that biomarkers can effectively discriminate subtypes of MI [2,14,15]. However, considering this situation is required because other diseases apart from MI may be suspected during the diagnosis process (especially non-ST elevation MI). In addition, type 2 MI and death can be caused by other complex parameters aside from those related to the cardiovascular system [13,55,56], which was also seen in the patients in the present study (Supplementary Table S1). However, type 1 MI has a high mortality rate due to cardiovascular disease, and many patients receive cardiac angiography [57,58]. In other words, type 1 patients are at high risk of death from MI; thus, proper classification and treatment are urgently needed. The markers obtained in this study can help distinguish type 1 MI based on their high specificity. However, they are not heart-derived markers and are also being studied as markers in other diseases, such as cancer. Therefore, they can be used as a complement to troponin, rather than as unique markers of MI.

There are several limitations to our study. First, the number of patients in the study is too small. Further studies with larger populations of participants are needed to validate the results of this study. For instance, the outliers eliminated in this small-scale study may not have been eliminated in a large-scale study. Second, the control group did not have enough diversity to represent all the troponin-elevated diseases (excluding type 1 MI). Type 2 MI, for two phenomena, contributed over 80% (46% + 36%) of the total sample group. In the future, additional studies that include more troponin-elevated diseases will be required to confirm and validate the findings. Third, the diagnostic effect of using the discovered marker with troponin was not verified. This should be included in future studies to prove that the markers identified in this study can complement troponin and serve as a type 1 MI-specific marker.

## 4. Materials and Methods

### 4.1. Study Population

Samples were collected from the Eulji University Hospital between May 2021 and January 2022. The patients included in this study visited the hospital for chest pain or underwent a cardiac troponin assay owing to suspected MI during their hospitalization. All patients were classified using the overall results of an ECG, cardiac troponin test, and coronary angiography. In coronary angiography, patients were classified as type 1 MI when lesions caused by plaque rupture or coronary thrombosis were observed. In contrast, an interventional cardiologist classified patients showing incomplete vascular stenosis as “Others” (other disorders in which troponin levels are elevated, excluding type 1 MI) according to the fourth universal definition. All patient blood samples were collected during coronary angiography within 48 h of admission; however, for patients classified as having type 2 MI (mechanism associated with oxygen imbalance), blood samples were collected within 48 h of a cardiac troponin assay during hospitalization. Healthy controls without cardiovascular diseases were also included.

All blood samples were collected using a vacutainer without anticoagulant, stored at 25 °C for 2 h, and centrifuged at 4000× *g* for 5 min to obtain the serum.

### 4.2. High-Abundance Protein Depletion

All samples were mixed at a ratio of 1:3 buffer A, filtered into a Spin-X centrifuge tube filter (Corning, New York City, NY, USA), and centrifuged at 12,000× *g* for 1 min. Subsequently, highly abundant serum proteins were depleted using a multiple-affinity removal system (MARS) column (human 6, 4.6 × 50 mm; Agilent Technologies, Santa Clara, CA, USA) using an Agilent 1200 high-performance liquid chromatography (HPLC) system (Agilent Technologies, Santa Clara, CA, USA). The data library was generated by pooling all the disease samples and removing high-abundance proteins in the same manner.

### 4.3. Low-Abundance Protein Digestion

The obtained low-abundance proteins were concentrated in a Nanosep filter (Pall Nanosep, Ann Arbor, MI, USA) at 12,000× *g*, and samples were dried using a speed vacuum. Samples were dissolved with lysis buffer (8 M urea and 0.1 M Tris–HCl (pH 8.5)). Protein concentration was quantified using bicinchoninic acid (BCA) assay, and the pooled and individual sample concentrations were adjusted to 1 mg and 100 µg, respectively. To reduce disulfide bonds, tris (2-carboxyethyl) phosphine (TCEP) (Pierce, Rockford, IL, USA) was added to a final concentration of 5 mM, and the mixture was incubated at 37 °C at 400 rpm for 30 min. To adjust the pH by 8.3, 50 mM of Tris–HCl and iodoacetamide were added into the final concentration at 15 mM for alkylation and incubated at 25 °C at 400 rpm for 1 h. Samples were diluted sevenfold with 50 mM Tris–HCl. Trypsin was added to the sample for tryptic digestion and incubated at 37 °C at 800 rpm for 15 h. To quench the reaction, 10% FA was added, lowering the pH to ≤3. Finally, the sample was desalted, and the following procedure was performed using C18 cartridges. The cartridge was washed with 100% methanol, 80% acetonitrile with 0.1% FA, and 0.1% FA. The total digest volume was loaded, washed seven times with 0.1% FA, and eluted with 50% acetonitrile with 0.1% FA. Elutes were dried in ScanSpeed 40 coupled with Teflon and resuspended in 0.1% FA prior to liquid chromatography–tandem mass spectrometry (LC-MS/MS).

### 4.4. Peptide Fractionation

The peptides of the pooled sample were separated into a 12-well-setup OFFGEL fractionator according to the isoelectric points.

### 4.5. LC-MS/MS Setup

Analysis was performed on a nano-liquid chromatography (LC) system, Ekspert nLC415 (Eksigent Technologies, Dublin, CA, USA), coupled with a triple-time-of-flight (TOF) 5600 mass spectrometer (AB SCIEX; Framingham, MI, USA). The individual samples or fractioned pooled samples were injected into the Eksigent ChromXP nanoLC trap column (0.5 mm × 350 μm; 3 μm; Eksigent Technologies), 2 μL at a time. The separation was achieved at a flow rate of 300 nL/min for a 95 min run time, and separated samples were eluted through an Eksigent ChromXP nanoLC column (150 mm × 75 μm; 3 μm; Eksigent Technologies) combined with a nanospray tip (PicoTip Emitter Silica Tip by New Objective, Woburn, MA, USA) at a flow rate of 3 μL/min for 7 min. Mobile phase A was water with 0.1% FA, and mobile phase B was acetonitrile with 0.1% formic acid (FA). The gradient of mobile phase B was 0 min to 5%, 70 min to 35%, 76–82 min to 90%, and 83–95 min to 5%. Autocalibration with 50 fmol of galactose was conducted after each of the three runs. The MS system was used in the positive ion mode with the following parameters: curtain gas pressure of 25 psi, ion spray voltage of 2300 V, interface heater temperature of 150 °C, and ion source gas pressure of 15 psi.

The 12 pooled samples (fractionated) were analyzed using a data-dependent acquisition (DDA) mode to create a spectral library. The scan mass range setting was 250–2000 *m*/*z* in the precursor ion scan mode and 100–2000 *m*/*z* in the product ion scan mode. The individual samples were analyzed using the data-independent acquisition (DIA)-based sequential window acquisition of all theoretical mass spectra method. In the DIA method, the isolation width was set to 20 Da (including 1 Da for window overlap) for a mass range of 250–2000 Da, with 53 overlapping windows.

### 4.6. Data Processing

The peptide identification was conducted using Protein Pilot v.5.0 software (AB SCIEX) with a Uniprot_human SwissProt database. The following search parameters were set with Triple TOF 5600 species, *Homo sapiens*, Cys alkylation (iodoacetamide), and digestion (trypsin; allowing for two missed cleavages). The ion library obtained using Protein Pilot was imported into Peakview 2.2 (SCIEX). The DIA data were processed with the following parameters: five peptides per protein, five transitions per peptide, <1% false-discovery-rate (FDR) threshold, and 99% peptide confidence threshold. The exported protein area value was then imported into MarkerView v.1.3.1 (AB SCIEX). The data were normalized using the total area sum. Welch’s *t*-test was calculated to obtain differentially expressed proteins between the test and control groups (disease) with a *p*-value < 0.05 and fold change ≥ 1.3. Furthermore, final candidates related to type 1 MI were selected in the reference study. We performed a literature search by combining keyword searches using PubMed. The keywords focused on the relationship with thrombosis, which is a singularity that can distinguish type 1 MI from other disorders. The data were displayed using a heatmap in MetaboAnalyts 5.0.

### 4.7. Peptide Selection for MRM

The following parameters were used to select target peptide lists for our multiple reaction monitoring (MRM) assay: (1) FDR < 1% in the overall sample; (2) exclude peptides with a missed cleavage site (-R, -K); (3) exclude peptides with modification sites; and (4) containing ≤ 20 mers amino acids. In addition, four known MI marker proteins were selected for peptides from the relevant literature.

### 4.8. Analysis of Parameter Settings and MRM Method Development

The quantitative analysis of selected proteins was performed using a triple-quadrupole linear ion-trap MS (AB SCIEX 5500 QTRAP) equipped with an electrospray ionization source. A sample (5 μL) was separated using an ACQUITY UPLC BEH C18 column (2.1 × 150 mm, 1.7 μm; Waters, Milford, MA, USA) connected to a Waters guard column, ACQUITY UPLC BEH C18 VanGuard pro-column (2.1 × 5 mm, 1.7 μm; Waters) for 30 min. Mobile phase A was water with 0.1% FA, and mobile phase B was acetonitrile with 0.1% FA. The following gradients were applied at a flow rate of 250 μL/min for a total run time of 30 min: an initial flow of 10% to 15% for 1 min, followed by a linear gradient of 15% to 40% in 20 min, 40% to 90% for 21 min, and then returning to 10% B for 25.5 min to 30 min for the same starting condition. The ionization source was operated in positive ion mode set to 30 psi for the curtain gas, an ion spray voltage of 5500 V, a temperature of 400 °C, 40 psi for nebulizer gas 1, and 60 psi for nebulizer gas 2.

The MS analysis parameter was optimized for synthetic peptides. The analysis methods were based on the parameters obtained using Skyline or manual optimization of Analyst software (version 1.7.1, SCIEX). During optimization, we selected a fragment ion with a high intensity when the precursor ion was split and selected the collision energy (CE) and declustering potential (DP) parameters that were most suitable for obtaining each ion. The entrance potential (EP) and collision cell exit potential (CXP) were fixed separately to positive values of 10 and 11 V. The three most intense transition parameters were selected, the standards were evaluated with the selected transition parameter method, and the retention time was obtained to create the scheduled MRM method. All MRM raw data were processed using Multiquant software 3.0.2 (SCIEX). Peak areas were integrated with a 1.5-point Gaussian smooth width; a 30 s retention time (RT) half window; and 3-point minimum peak width, 100-point minimum peak height, and 2-point peak splitting parameters. For the quantitative measurement of endogenous peptides, standard curves (1/x weighting) of each peptide were developed with a serial dilution of 5–7 calibration points. The synthesized internal standard (IS) used for normalization was injected at an equal final concentration (10 nM) into every sample. Calculated peptide concentrations were estimated using the peak-to-area ratio of endogenous peptides and IS peptides.

### 4.9. Statistical Analysis

Continuous variables with normal distributions were described as means and standard deviations, whereas non-normal distributions were described as medians and 25th and 75th percentiles. The unpaired *t*-test or Mann–Whitney U test was used for continuous variables. Furthermore, categorical variables were described as n (%), and the Fisher exact test was used to compare all characteristics between type 1 MI and type 2 MI. Likewise, the quantitative MRM data also used the unpaired *t*-test, Welch’s *t*-test, and Mann–Whitney test according to the normality test results and variance. In comparison between control and disease groups, the Kruskal–Wallis test followed by Dunn’s multiple comparisons test and the Brown–Forsythe test followed by Dunnett’s T3 multiple comparisons test were performed according to the results and variance of the normality test. The receiver-operating characteristic (ROC) curve was used to find the most effective biomarker. This allowed us to obtain the sensitivity, specificity, and cutoff values. All the data were analyzed using GraphPad Prism 8.4.2. Only the F-test to evaluate variance was performed in Microsoft Excel 2302 (Redmond, WA, USA).

## 5. Conclusions

In this study, we have identified three unique markers to effectively distinguish type 1 MI from other troponin-rising diseases. These markers, AGP2, CBG, and TREA, can help to diagnose type 1 MI and improve our understanding of the disease pathogenesis; however, further research is required for validation. The markers identified in this study may be present in other diseases related to thrombosis and plaque. Nonetheless, using these biomarkers in combination with other signs such as chest pain, ECG, and troponin levels during advanced MI diagnosis would enhance diagnostic accuracy.

## Figures and Tables

**Table 1:** Baseline characteristics.

	All (n = 60)	Discovery (n = 22)			Verification (n = 60)		
		T1MI (n = 12)	Others ^a^ (n = 10)	*p*-Value	T1MI (n = 32)	Others ^a^ (n = 28)	*p*-Value
Age (year)	73 (60.5, 80)	69.6 ± 11.6	70.1 ± 17.5	0.935	74 (62.0, 79.8)	72.5 (59, 80.8)	0.805
Male, n (%)	37 (61.7)	10 (83.3)	4 (40)	0.074	22 (68.8)	15 (53.6)	0.291
BMI, kg/m^2^	23.5 (21.5, 26)	23.1 ± 2.9	25.4 ± 2.6	0.044	23.4 (21.6, 25.6)	24.2 (21.5, 26.5)	0.800
Systolic BP, mmHg	130.4 ± 19.9	135.3 ± 14.4	133.5 ± 12.7	0.768	126.9 ± 18.1	134.4 ± 21.3	0.149
Diastolic BP, mmHg	78.5 (69, 89.8)	78.5 (71.3, 88.8)	79.5 (70, 84.8)	0.936	74 (67.5, 88.5)	80 (70, 90)	0.394
Risk Factors							
Past smoker	9 (15)	2 (16.7)	2 (20)	>0.999	4 (12.5)	5 (17.9)	0.721
Current smoker	13 (21.7)	4 (33.3)	0 (0)	0.096	13 (40.6)	4 (14.3)	0.043
Hypertension	32 (53.3)	5 (41.7)	7 (70)	0.231	16 (50.0)	16 (57.1)	0.613
Hyperlipidemia	19 (31.7)	3 (25)	5 (50)	0.377	9 (28.1)	10 (35.7)	0.586
Diabetes	25 (41.7)	3 (25)	6 (60)	0.192	11 (34.4)	14 (50.0)	0.296
CAD family history	6 (10)	1 (8.3)	1 (10)	>0.999	4 (12.5)	2 (7.1)	0.675
Past Medical History							
Previous myocardial infarction	5 (8.3)	1 (8.3)	1 (10)	>0.999	3 (9.4)	2 (7.1)	>0.999
Previous heart failure	19 (31.7)	3 (25)	2 (20)	>0.999	10 (31.3)	9 (32.1)	>0.999
Previous revascularization	9 (15)	2 (16.7)	1 (10)	>0.999	6 (18.8)	3 (10.7)	0.192
ECG							
ST-depression	15 (25)	5 (41.7)	0 (0)	0.040	12 (37.5)	3 (10.7)	0.020
ST elevation	18 (30)	6 (50)	1 (10.0)	0.074	14 (43.8)	4 (14.3)	0.007
Nonspecific change	27 (45)	1 (8.3)	9 (90.0)	0.0003	6 (18.8)	21 (75.0)	<0.0001
Laboratory Findings							
Hemoglobin, g/dL	12.6 ± 2	13.19 ± 2	11.9 ± 2.1	0.186	12.9 ± 2	12.1 ± 2	0.115
Peak troponin, ng/mL	0.9 (0.3, 2.5)	1.4 (0.5, 2.1)	0.3 (0.2, 0.5)	0.014	2.7 ± 2.6	1.90 ± 1.2	0.002
CK-MB, ng/mL	23 (7.7, 64.9)	31.7 (10.8, 104.7)	7.3 (5.0, 18.8)	0.009	51.0 (10.7, 137.6)	13.5 (5.6, 36.1)	0.002
CRP	0.9 (0.3, 3.1)	0.9 (0.2, 3.4)	2.4 (0.2, 5.3)	0.528	0.6 (0.3, 2.5)	1.3 (0.7, 3.4)	0.178

Data are expressed as n (%) for categorical variables. Data are expressed as median (interquartile range) or n (%) as continuous variables. ^a^ Others: diseases with elevated cardiac troponin other than type 1 myocardial infarction. Abbreviations: T1MI, type 1 myocardial infarction; CAD, cardiac artery disease; BP, blood pressure; CK-MB, creatine kinase MB isoenzyme.

**Table 2:** MRM parameters of the data acquisition method for peptides.

Protein	Peptide	Transition	RT (min)	CE	DP
α-1-acid glycoprotein 1	YVGGQEHFAHLLILR	877.0/235.3	7.3	45.0	74.6
α-1-acid glycoprotein 2	EHVAHLLFLR	617.9/267.1	6.3	76.2	35.1
Apolipoprotein B-100	GFEPTLEALFGK	654.9/205.1	12.8	78.9	29.4
Apolipoprotein C-I	TPDVSSALDK	516.8/466.2	3.3	68.8	23.5
Cathelicidin antimicrobial peptide	FALLGDFFR	543.3/219.0	14.1	82.4	31.9
Corticosteroid-binding globulin	GTWTQPFDLASTR	740.4/906.5	8.5	85.1	37.5
Fibronectin	WLPSSSPVTGYR	675.4/525.7	5.7	80.3	30.2
Prenylcysteine oxidase 1	LFLSYDYAVK	609.8/261.1	8.3	75.6	25.8
Serum amyloid oxidase 1	EALQGVGDMGR	566.7/691.3	3.5	72.4	30.3
Serotransferrin	EGYYGYTGAFR	642.3/551.4	5.0	79.5	47.4
CD40 ligand	SQFEGFVK	471.2/249.2	4.6	77.8	41.3
Matrix metallopeptidase 9	AVIDDAFAR	978.2/465.2	4.9	216.0	63.0
Myeloperoxidase	IANVFTNAFR	576.8/755.4	8.4	73.2	30.6
Pregnancy-associated plasma protein A	EQVDFQHHQLAEAFK	458.2/449.2	4.2	90.6	17.0

Abbreviations: CE, collision energy; DP, declustering potential; RT, retention time.

**Table 3:** Summary of the comparison of the diagnostic capabilities of markers.

Protein	Sensitivity	Specificity	Cut-off Value (nM ^a^)
α-1 acid glycoprotein 2	90.63 (75.8–96.8)	89.3 (72.8–96.3)	<337.3
Corticosteroid-binding globulin	86.7 (70.3–94.7)	85.7 (68.5–94.3)	<12.4
Serotransferrin	84.4 (68.3–93.1)	85.7 (68.5–94.3)	<0.4

Data are expressed as % (95% confidence interval). ^a^ nM: nanomolar.

## Data Availability

All data generated or analyzed are included in the current manuscript and are available from the corresponding author upon reasonable request.

## Supplementary Materials

The following supporting information can be downloaded at: https://www.mdpi.com/article/10.3390/ijms24098097/s1.
