# Supplementary material for: Potential Biomarkers to Distinguish Type 1 Myocardial Infarction in Troponin-Elevated Diseases
Source: Int J Mol Sci. 2023 Apr 30;24(9):8097. doi: 10.3390/ijms24098097 (PMC10179038; doi:10.3390/ijms24098097)
Supplement: Supplementary file 1 [file ijms-24-08097-s001.zip › ijms-2334855-supplementary.pdf]

Supplementary Table S1. Classification of patients in the Others group

Classification [a]	n	Classification [b]	n
Type 2 MI (O_2 imbalance)	13 (46.4)	Pneumonia	4 (31)
		Aortic stenosis	2 (15)
Type 2 MI (Coronary artery spasm)	10 (35.7)	Serious bradycardia	2 (15)
		Atrial tachycardia, hypotension	2 (15)
Myocarditis	3 (10.7)	Atrial fibrillation with rapid heartbeat	1 (8)
Perimyocarditis	1 (3.6)	Chronic obstructive pulmonary disease	1 (8)
Stress-induced cardiomyopathy	1 (3.6)	Scrub typhus	1 (8)

Data are expressed as n (%) for categorical variables.

[a] Classification: causes of troponin release in Others group.

[b] Classification: distinct causes of O_2 imbalance among the causes of Type 2 MI.

Others, diseases with elevated cardiac troponin other than type 1 myocardial infarction.

Supplementary Table S2. T-test results for candidate proteins

No.	Accession number [a]	Protein	Fold change	p-value
1	P02763	Alpha-1-acid glycoprotein 1	0.2902	0.0008
2	P08603	Complement factor H	0.4533	0.0000
3	Q9HDC9	Adipocyte plasma membrane-associated protein	0.5108	0.0056
4	P08185	Corticosteroid-binding globulin	0.5987	0.0022
5	P02746	Complement C1q subcomponent subunit B	0.6352	0.0001
6	P02787	Serotransferrin	0.6574	0.0096
7	P19652	Alpha-1-acid glycoprotein 2	0.6732	0.0440
8	P02747	Complement C1q subcomponent subunit C	0.6933	0.0029
9	P00736	Complement C1r subcomponent	0.7380	0.0055
10	P01023	Alpha-2-macroglobulin	0.7722	0.0042
11	P04114	Apolipoprotein B-100	1.3201	0.0406
12	P07357	Complement component C8 alpha chain	1.3249	0.0037
13	P35542	Serum amyloid A-4 protein	1.3356	0.0211
14	P02655	Apolipoprotein C-II	1.3672	0.0476
15	P04217	Alpha-1B-glycoprotein	1.3929	0.0136
16	P80108	Phosphatidylinositol-glycan-specific phospholipase D	1.4520	0.0191
17	P12259	Coagulation factor V	1.4577	0.0266
18	P02743	Serum amyloid P-component	1.5395	0.0098
19	P01008	Antithrombin-III	1.5750	0.0014
20	P43121	Cell surface glycoprotein MUC18	1.6072	0.0492
21	Q9UHG3	Prenylcysteine oxidase 1	1.6378	0.0130
22	Q01459	Di-N-acetylchitobiase	1.8879	0.0134
23	P22105	Tenascin-X	2.0128	0.0291
24	P01591	Immunoglobulin J chain	2.1209	0.0454
25	P02751	Fibronectin	2.3311	0.0089
26	P49908	Selenoprotein P	2.4414	0.0363

No.	Accession number [a]	Protein	Fold change	p-value
27	P02654	Apolipoprotein C-I	3.1799	0.0000
28	P62328	Thymosin beta-4	4.4752	0.0007
29	P49913	Cathelicidin antimicrobial peptide	5.2211	0.0008

[a] UniProtKB accession numbers.

Supplementary Table S3. Baseline characteristics of healthy controls. .

	Age (year)	Male, n (%)	BMI, kg/m^2	Hypertension		Hyperlipidemia		Diabetes		Heart disease [a]	
				Past	Current	Past	Current	Past	Current	Past	Current
All (30)	59.4 ± 4.9	15 (50)	23.8 (22.3, 26.2)	0 (0)	0 (0)	2 (6.7)	0 (0)	0 (0)	0 (0)	0 (0)	0 (0)

Data are expressed as n (%) for categorical variables.

Data are expressed as median (interquartile range) or n (%) for continuous variables.

[a] Heart disease: myocardial infarction and angina pectoris

Captions for illustrations

Supplementary Figure S1. Comparison of disease and healthy control groups. The graphs display the protein expression patterns of the diseased (T1MI and Others) and non-diseased (Healthy) groups. The three groups were analyzed by Brown–Forsythe ANOVA or Kruskal–Wallis tests. (A)α-1 acid glycoprotein 1 level was significantly different between T1MI and healthy controls. (B)α-1 acid glycoprotein 2 was significantly different between disease and healthy controls. (C) Cathelicidin antimicrobial peptide was significantly different between T1MI and healthy controls. (D) CD40 ligand was significantly different between disease and healthy controls. (E) Corticosteroid-binding globulin was significantly different between T1MI and healthy controls. (F)Serotransferrin was significantly different between disease and healthy controls.

*, $p < .05$; **, $p < .005$; ***, $p < .0001$.

Others, diseases with elevated cardiac troponin other than type 1 myocardial infarction.

Healthy, healthy controls without cardiovascular disease.

Abbreviation: T1MI, type 1 myocardial infarction.

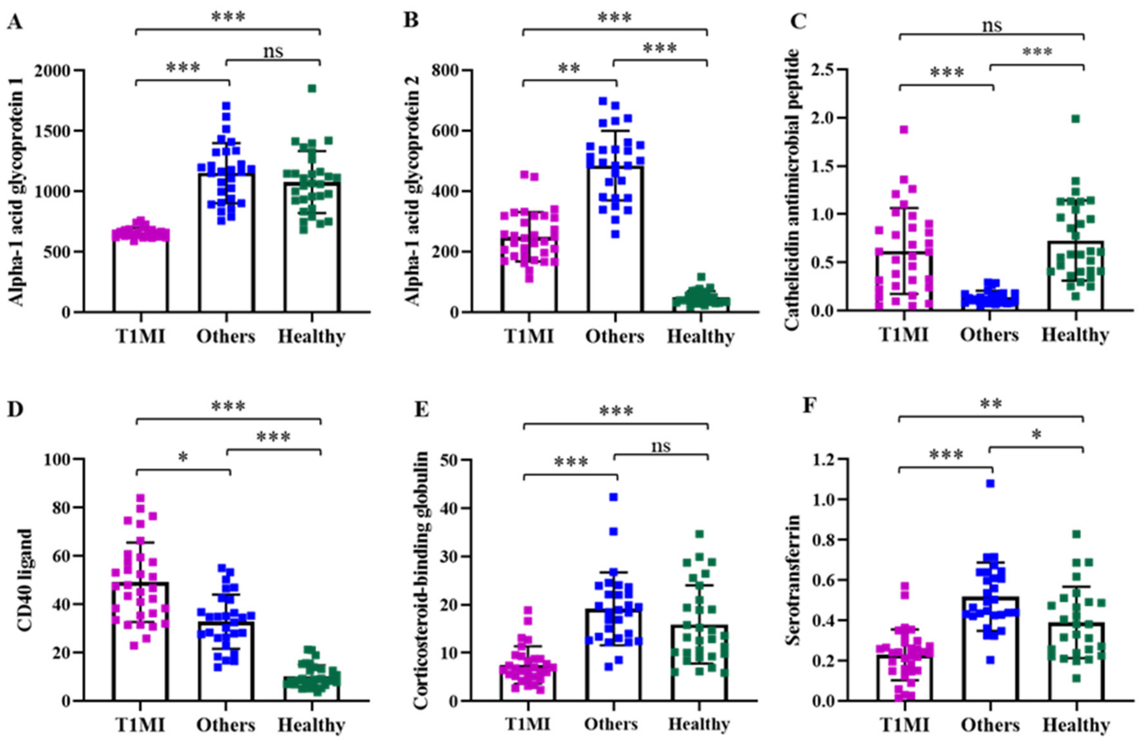

Supplementary Figure S1.
